# Hollow MFI Zeolite Supported Pt Catalysts for Highly Selective and Stable Hydrodeoxygenation of Guaiacol to Cycloalkanes

**DOI:** 10.3390/nano9030362

**Published:** 2019-03-04

**Authors:** Xiaopo Niu, Fuxiang Feng, Gang Yuan, Xiangwen Zhang, Qingfa Wang

**Affiliations:** 1 Key Laboratory for Green Chemical Technology of Ministry of Education, School of Chemical Engineering and Technology, Tianjin University, Tianjin 300072, China; xiaoponiu@tju.edu.cn (X.N.); 1014207069@tju.edu.cn (F.F.); gangyuan@tju.edu.cn (G.Y.); zhangxiangwen@tju.edu.cn (X.Z.); 2 Collaborative Innovation Center of Chemical Science and Engineering (Tianjin), Tianjin University, Tianjin 300072, China

**Keywords:** hollow MFI zeolite, Pt catalyst, guaiacol, hydrodeoxygenation, cycloalkane

## Abstract

Hollow Silicalite-1 and ZSM-5 zeolites with hierarchical porous shells have been synthesized by using a dissolution-recrystallization method. The morphology, structure, and acidity of these zeolites supported Pt catalysts were characterized by XRD, FT-IR, MAS-SSNMR, FE-SEM, FE-TEM, N_2_-BET, XPS, NH_3_-TPD, and CO pulse chemisorption. Compared to the conventional ZSM-5 supported Pt catalyst, the special structure in hollow ZSM-5 zeolite significantly promotes the dispersion of metallic Pt and the synergistic effect between metal active sites and acid sites. These boost the catalytic activity, selectivity of guaiacol hydrodeoxygenation toward cycloalkanes and long-term stability over the Pt/hollow ZSM-5 catalyst combined with improved mass transfer of products and reactants derived from the hierarchical hollow porous structure. Moreover, the Pt/hollow ZSM-5 catalyst exhibits excellent low temperature catalytic activity to completely transform guaiacol into cycloalkanes with the cyclohexane selectivity of more than 93% at 220 °C, suggesting that hollow ZSM-5 zeolite is a promising support for upgrading of bio-oils.

## 1. Introduction

Developing renewable and sustainable transportation liquid fuels has attracted significant attention due to the gradual depletion of fossil resources and stringent environmental regulation [1,2]. Among the various sustainable energy sources, biomass is the only renewable organic carbon resource in nature, which endows it with unique advantages in producing a variety of value-added chemicals and fuels [3,4]. Bio-oils, from fast pyrolysis of biomass, are considered as the most promising alternative energy. However, bio-oils contain many oxygenic compounds such as carboxylic acids, carbohydrates, phenols, furans, sugars, aldehyde, ketones, and water [5,6]. Consequently, the high oxygen content (35–50 wt%) leads to deteriorated properties such as poor heating value, high viscosity, corrosiveness, low caloric value, low thermal and chemical stabilities, and immiscibility with conventional fossil fuels [7,8]. As a result, bio-oils must be upgraded before they can be used as standard liquid fuels. Catalytic hydrodeoxygenation (HDO) is generally considered as the most effective strategy for upgrading bio-oils [9].

Various catalysts have been developed and used in the HDO of bio-oils to produce hydrocarbons. In earlier studies, conventional hydrodesulfurization (HDS) catalysts such as supported NiMoS [10] and CoMoS [11] catalysts were employed to remove oxygen from bio-oils and showed high deoxygenation activity. However, these catalysts suffer from gradual deactivation during the HDO reaction due to the loss of sulfur, because the sulfur content in bio-oils is very low and the lost sulfur will contaminate the resulting fuels [5]. Hence, non-sulfide catalysts have been developed and investigated including noble metals [12,13,14,15,16], transition metal phosphides [9,17,18,19], carbides [20,21], and nitrides [22,23] in order to replace sulfided catalysts and these catalysts exhibited excellent HDO activity of bio-oils and deoxygenation selectivity for hydrocarbons. However, the rapid deactivation because of coke formation during the HDO reaction hinders their extensive application [24,25]. It is clear that catalyst support plays a crucial role in HDO of bio-oils, Al_2_O_3_ [26,27], TiO_2_ [28,29,30], ZrO_2_ [31,32], SiO_2_ [25,33,34], zeolite (e.g., ZSM-5, Beta, HY) [18,35,36,37,38,39,40,41], and activated carbon [42,43] have been investigated in HDO of bio-oils, and the zeolite supported noble metal catalyst exhibited better catalytic HDO performance. Unfortunately, the diffusion of reactants and products is usually limited by the microporous structure of zeolite, which decreases the catalytic activity and stability of catalyst. Fabricating intra-crystalline mesoporosity or macroporosity and/or decreasing the size of zeolite crystals are effective methods to overcome the mass transfer limitation and to boost catalyst stability [44,45,46]. Hunns et al. have investigated hierarchical mesoporous ZSM-5 supported Pd for HDO of *m*-cresol and found that the hierarchical porous structure enhanced the dispersion of metallic Pd, and significantly improved *m*-cresol conversion [39]. Wang et al. demonstrated that Pt supported on mesoporous ZSM-5 exhibited better performance in dibenzofuran HDO reaction than Pt supported on conventional microporous ZSM-5 [47]. These results indicate that the hierarchical porous structure significantly promotes the catalyst activity and stability for bio-oils upgrading. However, it still is a great challenge and highly desired to design and fabricate novel hierarchical catalysts with controllable selectivity and stability.

In recent years, hollow zeolites with a cavity in core and hierarchical porous in shell have attracted much attention due to their high surface area, thermostability, good diffusivity, and excellent shape-selective catalytic capabilities [48,49,50,51,52]. Wu et al. synthesized hollow TS-1 zeolite with mesoporous structure for cyclohexanone ammoximation to improve the activity and stability of catalyst [51]. Zhou et al. demonstrated that bimetallic PtSn supported on hollow silica spheres had excellent activity for acetic acid hydrogenation duo to more Pt active sites exposed on the surface of hollow silica [50]. Wang et al. reported that Mo supported on hollow ZSM-5 catalyst exhibited higher CH_4_ conversion and aromatic selectivity as well as longer lifetime compared with conventional Mo/ZSM-5 catalyst in methane dehydroaromatization reaction [48]. Therefore, hollow structure zeolites are expected to exhibit good performance in bio-oil upgrading. However, rare work has been carried out.

Phenols are the primary oxygenates in bio-oils and phenolic oxygen is very difficult to be removed [9,35]. Therefore, guaiacol, a typical lignin monomer containing both methoxy and phenolic hydroxyl groups, is usually selected as a model compound to evaluate the HDO performance of various catalysts. In this context, we investigate the selective HDO of guaiacol over the hollow MFI structure zeolite supported Pt catalysts. Hollow Silicalite-1 and ZSM-5 zeolites are synthesized by using a dissolution-recrystallization strategy. Additionally, Pt catalysts supported on various MFI zeolites are prepared by the incipient wetness impregnation method. The role of introducing hollow structure in MFI zeolite supports catalytic performance of Pt catalysts is mainly concerned.

## 2. Experimental Section

### 2.1. Catalyst Preparation

Synthesis of Silicalite-1 (S-1) zeolite: The parent S-1 zeolite was synthesized by conventional hydrothermal synthesis method using a starting molar composition of 1 TEOS (tetraethyl orthosilicate): 0.3 TPAOH (tetrapropylammonium hydroxide): 39 H_2_O. In a typical run, 16.0 g of TEOS ( Aladdin, 99 %), 39.5 g of H_2_O, and 18.6 g of TPAOH ( innochem, 25 wt% in water) was mixed and stirred at room temperature for 12 h to ensure complete TEOS hydrolysis. The gel was then transferred into a 100 mL Teflon-lined steel autoclave and heated at 170 °C for 72 h. After cooled to room temperature, the product was then recovered by centrifugation, washed with distilled water and dried at 110 °C overnight. Finally, it was calcined at 550 °C in air for 6 h.

Synthesis of hollow silicalite-1 (hS-1) zeolite: The hS-1 zeolite was prepared by a dissolution-recrystallization strategy using S-1 zeolite as the precursor [53,54]. Typically, 4 g of calcined S-1 zeolite dispersed in 80 mL of 0.5 M TPAOH solution. The mixture was stirred for 5 min and then transferred into a Teflon-lined autoclave, heating at 170 °C under static conditions for 24 h. The solution was cooled down, recovered, washed with distilled water, dried at 110 °C overnight, and then calcined in air at 550 °C for 6 h.

Synthesis of hollow ZSM-5 (hZSM-5) zeolite: The synthetic approach of hZSM-5 zeolite is similar to that for hS-1 zeolite. 80 mL of TPAOH solution and 0.1261 g of aluminum nitrate nonahydrate (Al(NO_3_)_3_·9H_2_O, ACROS, 99%) were mixed and stirred for 30 min, then 4 g of calcined S-1 added. After an additional 5 min stirring, the mixture was treated at 170 °C in a Teflon-lined autoclave for 24 h. Finally, the hZSM-5 zeolite was recovered and calcined in the same procedure with hS-1 zeolite.

Preparation of supported Pt catalysts: Pt supported on the as-synthesized zeolite catalysts were prepared by incipient wetness impregnation (IWI) method. Typically, 5 g of zeolite support was impregnated with H_2_PtCl_6_·6H_2_O (Aladdin, 99%) aqueous solution containing a predetermined quantity of Pt to achieve the final Pt loading of 1 wt%. The impregnated sample was then kept overnight at room temperature and dried at 110 °C for 12 h. Finally, the sample was calcined at 450 °C for 4 h. As a comparison, conventional ZSM-5 (cZSM-5) zeolite (Si/Al ratio of 80, Nankai University Catalyst Co., Ltd., Tianjin, China) was also used as the support to prepare the Pt catalyst. The obtained catalysts were denoted as Pt/S-1, Pt/hS-1, Pt/hZSM-5, and Pt/cZSM-5, respectively.

### 2.2. Catalyst Characterization

Powder X-ray diffraction (XRD) patterns were recorded on a Rigaku D/MAX-2500 diffractometer (Rigaku, Ltd., Tokyo, Japan) using Cu Kα radiation using nickel-filtered Cu Kα X-ray source (40 kV, 200 mA, λ = 1.5406 Å) at a scanning rate of 0.02° over the range between 5° and 90°. Fourier transformed infrared (FT-IR) spectra were recorded on a Bruker VERTEX 70 spectrometer (Bruker Ltd., Karlsruhe, Germany) in a wavenumber range between 4000 and 400 cm^−1^ with a resolution of 4 cm^−1^. Magic angle spinning solid-state nuclear magnetic resonance (MAS-SSNMR) spectra were obtained on a Varian Infinityplus 300 spectrometer (Varian Ltd., Palo Alto, America). Field emission scanning electron microscopy (FE-SEM) images were obtained on a Hitachi S-4800 scanning electron microscope (Hitachi Ltd., Tokyo, Japan) at 5 kV. Field emission transmission electron microscopy (FE-TEM) was carried out on a JEM-2100F electron microscope (JEOL, Tokyo, Japan) with an accelerating voltage 200 kV. Nitrogen adsorption and desorption isotherms (N_2_-BET) were obtained on a Micromeritics ASAP 2460 analyzer (Micromeritics Ltd., Georgia, America). Before the measurement, 0.15 g of the samples were degassed under vacuum at 300 °C overnight. The Brunauer–Emmett–Teller (BET) equation was applied to calculate the total specific surface area, while the pore volume and specific area of micropore were calculated by using t-plot method. The pore size distribution curves were derived using the non-local density functional theory (NLDFT) model. The Si/Al molar ratio was measured by inductively coupled plasma-optical emission spectrometer (ICP-OES) (Agilent 7700x, Agilent Ltd., California, America).

X-ray photoelectron spectra (XPS) measurements were performed on Thermo ESCALAB 250XI (Thermo Fisher Scientific, Massachusetts, America) with Al Kα X-ray radiation for the X-ray source. The samples were reduced under catalytic conditions and exposure to air minimized prior to analysis. For energy calibration, the C1s binding energy at 284.8 eV was taken as a reference value. Temperature programmed desorption of ammonia (NH_3_-TPD) and CO pulse chemisorption of all the samples were measured on an AutoChem1 II 2920 (Micromeritics Ltd., Georgia, America) apparatus equipped with a thermal conductivity detector (TCD). For NH_3_-TPD measurement, 0.15 g of the sample was pretreated in helium (He) at 450 °C for 1 h and then cooled to 100 °C. A mixture of 10% NH_3_ in He was absorbed at 100 °C for 40 min and purged with pure He at the same temperature for 2 h. The sample was heated and the desorption profile was recorded. In CO chemisorption experiments, the sample was pretreated in He at 300 °C for 1 h, reduced in H_2_ at 450 °C for 2 h, evacuated at 450 °C for 1 h, and then cooled to 40 °C in vacuum. Then, the CO adsorption isotherm was recorded at 50 °C based on the amount of adsorbed CO at different pressures. A CO to surface Pt atom stoichiometry of 1.0 was used in metal dispersion calculations [55].

### 2.3. Catalytic Evaluation

The catalytic HDO of guaiacol over different zeolites supported Pt catalysts were performed in a fixed-bed reactor with 6 mm inner diameter. In a typical run, 1.5 g of catalyst (20–40 mesh) was loaded in the center of reactor. The reaction temperature was controlled by three thermocouples on the reactor wall and monitored with a thermocouple directly placed in the catalyst bed. Prior to the experiment, the Pt catalyst was reduced in situ at 450 °C for 4 h under the H_2_ atmosphere and then cooled down to the reaction temperature. A solution of 5 wt% guaiacol in *n*-dodecane was used as the feedstock and supplied at a flow of 0.2 mL·min^−1^ using a high-pressure pump. The HDO reaction was performed at the temperature range from 220 to 280 °C, under a total pressure of 3 MPa and H_2_ flow rate of 100 mL·min^−1^. The weight hourly space velocity is 0.3064 h^−1^. The liquid products at different temperatures were collected for 2 h after the reaction reached the desired temperature and 1wt% n-tetradecanein was added as the internal standard. Then, the liquid samples were analyzed off-line with a Shimadzu GC-MS QP2020 (Shimadzu Ltd., Kyoto, Japan) using a commercial Rtx-5MS (50 m × 0.25 mm × 0.25 μm) column.

The guaiacol conversion (X_gua_) and product selectivity (S_product-*i*_) was calculated as follows: X_gua_ = (Mol_gua,in_ − Mol_gua_,_out_)/(Mol_gua_,_in_), S = (Mol_product-*i*_)/(Σ(Mol_products-*i*_)), where Mol_product-*i*_ is the mole number of product-*i* in the collected liquid sample.

## 3. Results and Discussion

### 3.1. Catalyst Properties

**Figure 1:**
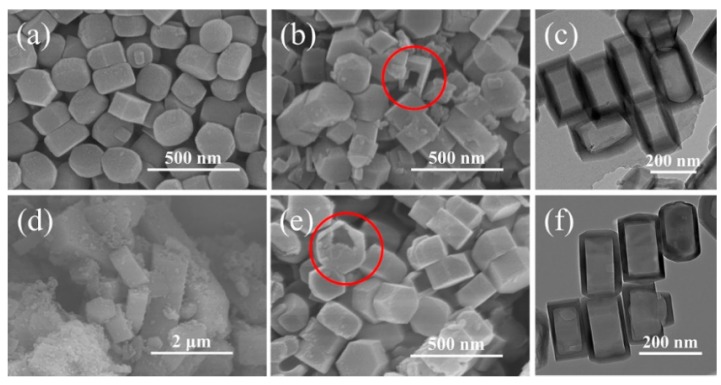
FE-SEM images of (**a**) S-1 (**b**) hS-1, (**d**) cZSM-5, (**e**) hZSM-5, and FE-TEM images of (**c**) hS-1 and (**f**) hZSM-5 zeolites.

The FE-SEM images of different zeolites are shown in Figure 1. It clearly showed that S-1, hS-1 and hZSM-5 zeolites exhibited similar morphology of ellipsoids with the size of about 210 nm. The parent S-1 zeolite had a little rough surface but a smooth one for hS-1 and hZSM-5 zeolites. Amorphous silica and some broken crystals with large cavities were also observed in the hS-1 and hZSM-5 zeolites and the crystal particles of these zeolites were a little larger than that of parent S-1. These results confirmed that the dissolution of crystal core of S-1 zeolite and recrystallization of dissolved species on the surface occurred in the presence of TPAOH at 170 °C, agreeing with the literature [53,54]. The FE-SEM images (Figure 1b,e) of the broken crystals definitely revealed that the hollow structure was formed for hS-1 and hZSM-5 zeolites. The TEM images (Figure 1c,f) further confirmed that these zeolites were composed of the similar hollow nanocrystals. In addition, the sizes of the hollow cavities were about 100 nm and the shell thickness of the zeolite crystal was about 20 nm. The introduction of aluminum species during the dissolution-recrystallization process had no effect on the formation of hollow structure. As a comparison, typical coffin-like morphology for cZSM-5 zeolite was obviously observed in Figure 1d and the zeolite crystals were the largest ones among the as-synthesized zeolites.

**Figure 2:**
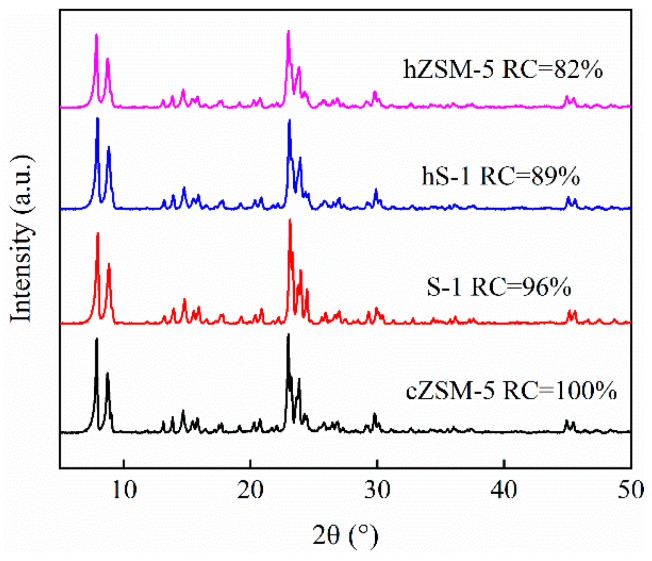
Powder XRD patterns of cZSM-5, S-1, hS-1, and hZSM-5 zeolites.

Figure 2 shows the powder XRD patterns of the as-synthesized S-1, hS-1, hZSM-5, and cZSM-5 zeolites. All the samples exhibited the five typical characteristic diffraction peaks at 7.98°, 8.84°, 23.12°, 23.95°, and 24.36°, indicative of the MFI-type zeolites [44]. This meant that the bulk crystal structures of hS-1 and hZSM-5 zeolites were maintained after dissolution-recrystallization process. This result was further confirmed by FT-IR spectra. As shown in Figure S1, all the zeolites showed the framework vibrations at 550 cm^−1^ and 452 cm^−1^, which are the characteristic bands of MFI-type zeolites [56]. The significant broad bands at 1230 cm^−1^, 1110 cm^−1^, and 800 cm^−1^ were attributed to the external asymmetric stretch, internal asymmetric stretch, and external symmetric stretch of typical high-silica zeolite [57,58]. Compared with parent S-1 zeolite, the relative crystallinity (RC) of hS-1 and hZSM-5 zeolites decreased by 7% and 14%, respectively, implying that a slight degradation of crystal framework occurred after dissolution-recrystallization treatment. The relative crystallinity of hZSM-5 was lower than that of hS-1, probably because aluminum species retarded the recrystallization of silica species dissolved from the parent S-1 [45]. The ^27^Al MAS-NMR spectra (Figure S2) of cZSM-5 and hZSM-5 zeolites showed a major resonance at about 53 ppm and a very weak resonance around 0 ppm, corresponding to tetrahedrally coordinated framework aluminum (AlO_4_) in silica frameworks and octahedrally coordinated non-framework aluminum (AlO_6_), respectively [54]. This confirmed that the Al species were essentially tetrahedrally coordinated after dissolution-recrystallization treatment.

**Figure 3:**
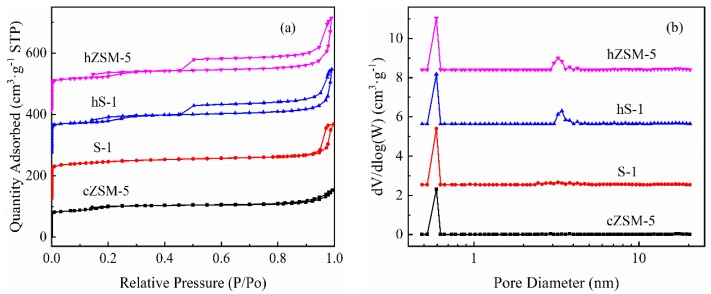
(**a**) N_2_ adsorption and desorption isotherms and (**b**) pore size distributions of different zeolites.

The nitrogen adsorption and desorption isotherms of different zeolites was further characterized and are shown in Figure 3. The hS-1 and hZSM-5 zeolites exhibited a pronounced H4 hysteresis loop at relative pressure P/P_0_ = 0.45 along with a sub-step around relative pressure P/P_0_ = 0.2, reflecting the existence of both micropore and mesoporous [59]. This result illustrated the generation of mesoporous in hollow MFI zeolites during TPAOH treatment process. The conventional ZSM-5 showed a type Ⅰ isotherm and no distinct hysteresis loop, which proved that cZSM-5 was a typical microporous zeolite without any mesoporous. The pore size distributions derived from non-local density functional theory (NLDFT) (Figure 3b) further confirmed the existence of micropore and mesoporous in hS-1 and hZSM-5 zeolites. Additionally, the mesoporous size was mainly located around 2–4 nm. The textual properties of various samples derived from N_2_ physisorption are summarized in Table 1. The cZSM-5 showed a typical specific surface area (S_BET_, 332 m^2^·g^−1^) and pore volume distribution of microporous zeolite. The parent S-1 zeolite had the similar textual structure with cZSM-5 zeolite except more developed micropores. The hS-1 and hZSM-5 zeolites had almost the same pore distribution. Compared with parent S-1, the mesoporous volume (V_meso_) increased by 79% and 80% for hS-1 and hZSM-5 zeolites, respectively. Interestingly, the specific surface area of hS-1 and hZSM-5 had a slightly decreased, while the total volume (V_total_) determined by t-plot method had no significant change. This could be attributed to the recrystallization leading to a smoother crystal surface and the formation of hierarchical porous hollow structure.

**Figure 4:**
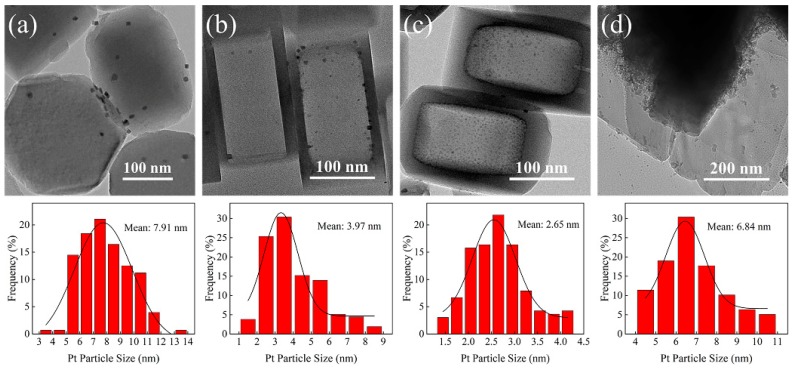
FE-TEM images and Pt particle size distributions of (**a**) Pt/S-1 (**b**) Pt/hS-1, (**c**) Pt/hZSM-5, and (**d**) Pt/cZSM-5 catalysts.

The size distributions of Pt particles supported on different zeolites were further analyzed and are illustrated in Figure 4. As shown in Figure 4a, the parent S-1 zeolite clearly showed an integral hexagonal crystal structure. Pt particles were unevenly distributed on the surface of S-1 zeolite due to the agglomeration. The mean size of Pt particles was estimated at about 7.91 nm. As for the Pt/hS-1 catalyst, Pt particles were evenly distributed on the surface (Figure 4b) with the particle size centered at 3.97 nm, much smaller than that of Pt/S-1 catalyst. This indicated that hierarchical hollow structure was favorable for improving the Pt dispersion. Moreover, the Pt/hollow ZSM-5 catalyst showed the smallest Pt particle size of 2.65 nm. The conventional Pt/cZSM-5 catalyst presented a mean size of Pt particles at 6.84 nm. These results indicated that the Pt dispersion was greatly boosted by the hollow structure and the introduction of framework aluminum could also promote the dispersion of metallic Pt, probably because more Pt particles adjacent to surface strong acid sites of the introduced Al center were formed to confine the allegation of Pt nanoparticles leading to a stronger metal-support interaction.

The variation of active metal dispersion on the support was confirmed by CO pulse chemisorption. As shown in Table 2, the CO uptakes of the different catalysts varied from 6.79 to 17.36 μmol·g^−1^. The metal dispersion of Pt/hS-1 and Pt/hZSM-5 increased significantly, suggesting that the hollow MFI structure promoted the Pt dispersion and more metal active sites were exposed on the surface. Microporous zeolites (S-1 and cZSM-5) are not conducive to the dispersion of metallic Pt. This was consistent with the TEM results (Figure 4).

**Figure 5:**
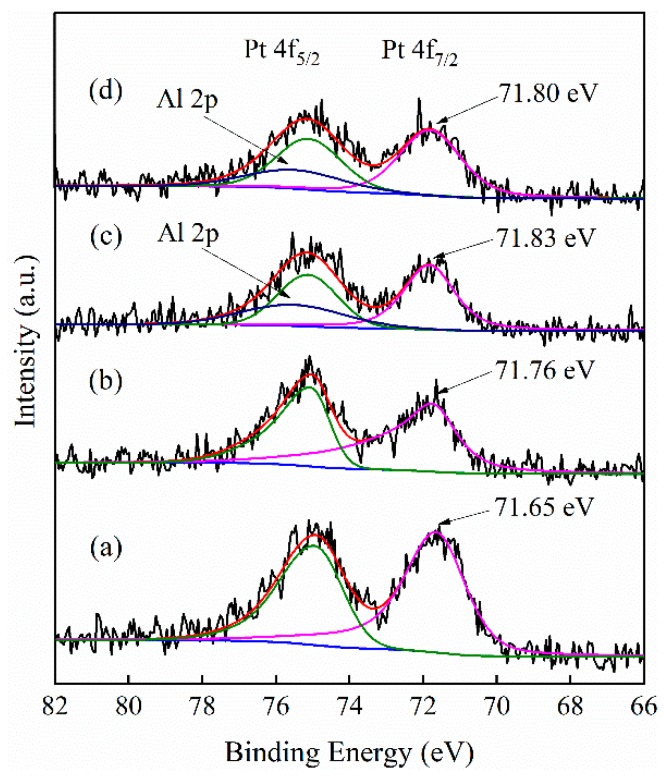
Pt 4f XPS spectra of (**a**) Pt/S-1, (**b**) Pt/hS-1, (**c**) Pt/hZSM-5, and (**d**) Pt/cZSM-5 catalysts.

In order to obtain a better understanding of the interaction between Pt and different MFI zeolite supports, the metal valence of Pt element in various catalysts were analyzed by XPS and the spectra are shown in Figure 5. After de-convolution of the spectra, these catalysts exhibited two energy bands at 71.65~71.83 and 74.95~75.13 eV (Figure 5 and Table S1), which are values for the Pt 4f 7/2 and 4f 5/2 electrons of metallic Pt [60,61]. The binding energy peaks of Pt 4f 5/2 and Pt 4f 7/2 are separated by 3.30 eV. This indicated that Pt particles in these catalysts existed in metallic state. Notably, compared with Pt/S-1, the binding energy of Pt 4f shifted 0.11 and 0.18 eV for Pt/hS-1 and Pt/hZSM-5, respectively. This could be attributed to the strong metal-support interaction [29,62], which was consistent with the results of Pt particle size distributions (Figure 4).

**Figure 6:**
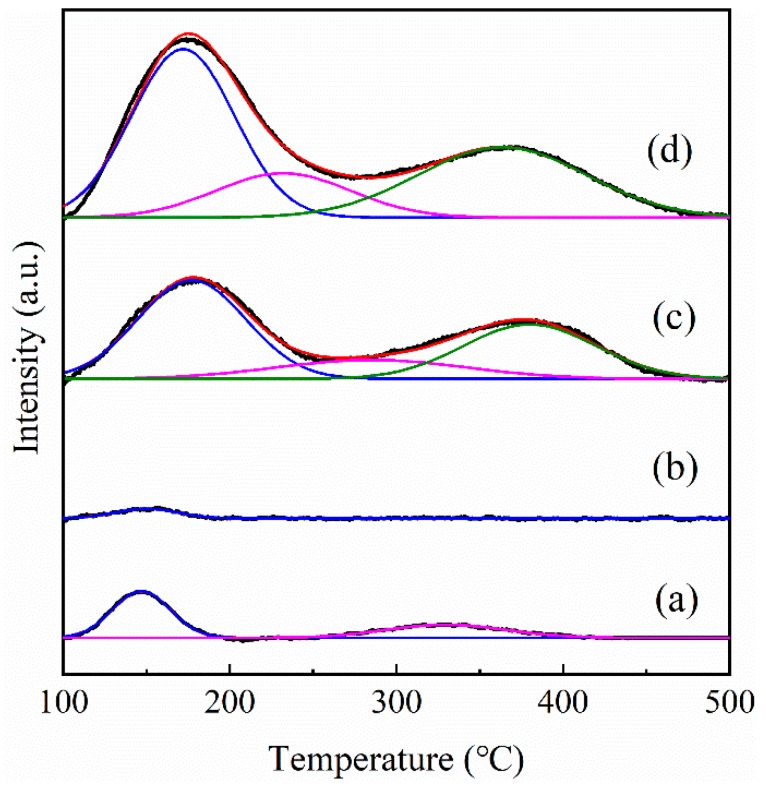
NH_3_-TPD profiles of (**a**) Pt/S-1, (**b**) Pt/hS-1, (**c**) Pt/hZSM-5, and (**d**) Pt/cZSM-5 catalysts.

The acidity of support has a significant effect on the catalytic activity for HDO of bio-oils [63,64]. The acidity distribution of all Pt catalysts characterized by NH_3_-TPD is shown in Figure 6. Three peaks around 172~177 °C, 232~285 °C and 329~380 °C were observed in the NH_3_-TPD profiles, which were attributed to weak, medium, and strong acidic sites, respectively. The quantitative analysis results are summarized in Table 2. Obviously, conventional ZSM-5 supported Pt catalyst exhibited the strongest acidity, attributed to the highest aluminum content in the zeolite according to the ICP-OES result (Table 2). The Pt/S-1 catalyst had very small amount of weak and strong acid sites, which mainly originated from silanol groups on the surface of parent S-1 [65,66]. The Pt/hS-1 had only weak acid sites with the smallest acid density of 7.08 μmol·g^−1^, which was decreased by 87% compared with Pt/S-1. The drastic acidity decrease for Pt/hS-1 might be caused by the destruction of the acidic sites on the surface of S-1 during TPAOH treatment. The acid sites density of Pt/hZSM-5 was significantly enhanced by introducing aluminum species compared with Pt/S-1 catalyst, because the dissolved amorphous silicon and aluminum species recrystallized on the surface of the parent S-1 to form the hierarchical nanosized ZSM-5 crystals. The total acidity of this catalyst was nearly half of Pt/cZSM-5 due to its higher Si/Al ratio, but the distribution of weak, medium, and strong acid sites was almost the same with the Pt/cZSM-5 catalyst (49%, 17%, and 34%, respectively).

### 3.2. Hydrodeoxygenation of Guaiacol

**Figure 7:**
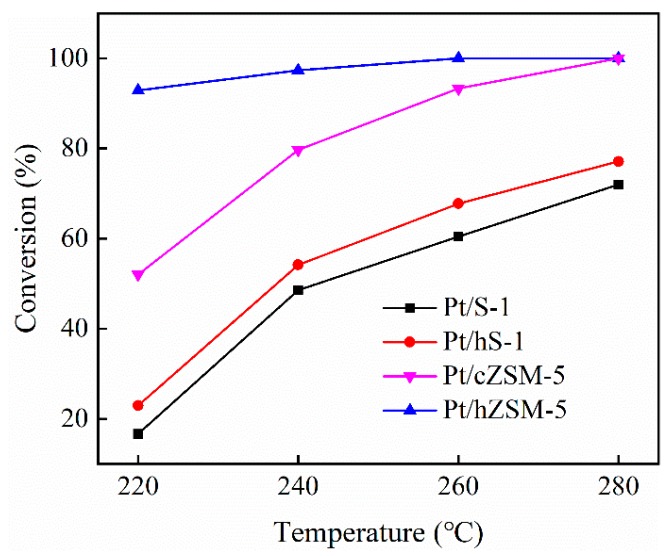
Conversion of guaiacol as a function of temperature over different Pt catalysts.

The catalytic performance of Pt catalysts supported on parent S-1, hollow S-1 (hS-1), hollow ZSM-5 (hZSM-5) and conventional ZSM-5 (cZSM-5) for HDO of guaiacol was tested in a fixed-bed reactor. The reaction was performed at 220 °C, 240 °C, 260 °C, and 280 °C under 3 MPa. Figure 7 shows the conversions of guaiacol over various Pt catalysts at different temperatures. The guaiacol conversion over the parent S-1 zeolite supported Pt catalyst was only 17% at 220 °C and up to 72% at 280 °C. When the hollow S-1 zeolite was used as the support, a higher guaiacol conversion increased by about 6% was obtained than that of Pt/S-1 catalyst at the same temperature. Similarly, the Pt/hZSM-5 also showed much higher conversion of guaiacol than Pt/cZSM-5. At a relatively low temperature, the activity enhancement over Pt/hZSM-5 catalyst became more significant (~40% at 220 °C vs. ~5% at 260 °C) compared with Pt/cZSM-5. The Pt/hZSM-5 catalyst exhibited the best activity with 100% guaiacol conversion at 260 °C and 100% selectivity to cycloalkanes even at low temperature of 220 °C. From Table 2, it could be observed that the acidity of hollow Pt/hS-1 and Pt/hZSM-5 catalysts was much lower than that of Pt/S-1 and Pt/cZSM-5 catalysts, respectively. Therefore, it could be concluded that the enhanced activity for Pt/hS-1 and Pt/hZSM-5 was mainly derived from the hollow structure, which promoted the dispersion of metallic Pt (Table 2) and the diffusion of reactants and products. In addition, it was found that the activity enhancement from Pt/hZSM-5 to Pt/cZSM-5 was much higher than that from Pt/hS-1 to Pt/S-1 at low temperature. Compared with Pt/hS-1, the increased acid sites for Pt/hZSM-5 mainly came from the introduced framework Al species. This indicated that high Al acid sites density of support was also conducive to improve the catalytic performance for guaiacol conversion. In addition, although the acidity of Pt/cZSM-5 was almost twice that of Pt/hZSM-5 (Table 2), more Pt active sites adjacent to strong Al acid sites were formed in the Pt/hZSM-5 catalyst to facilitate the diffusion of intermediates between the metal-support active sites. Therefore, Pt/hZSM-5 showed a higher guaiacol conversion than Pt/cZSM-5 at the same reaction temperature. These results indicated that the activity enhancement for Pt/hZSM-5 catalyst was attributed to the synergy of hollow structure and metal-acid sites interaction, in which the higher dispersion of Pt metal on the surface of hollow ZSM-5 support was achieved (Figure 4c) and the diffusion of reactants and products was promoted by the hierarchical hollow structure.

**Figure 8:**
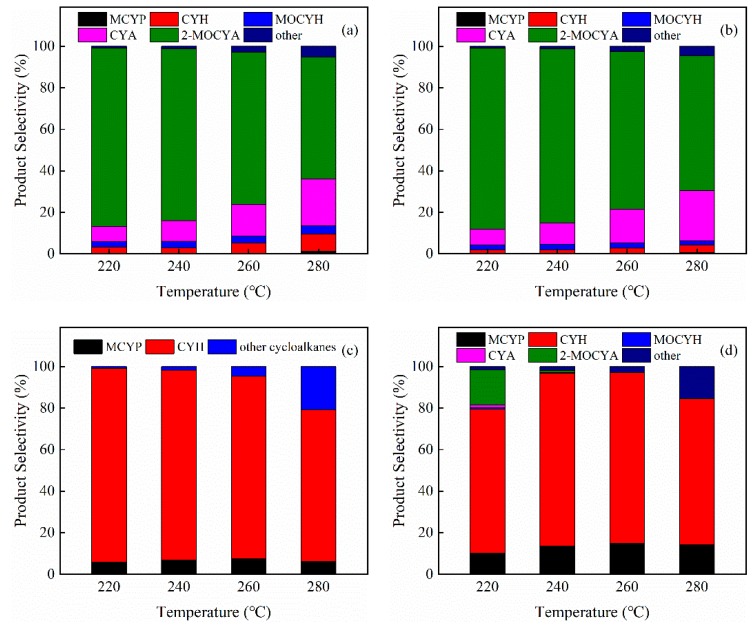
Product selectivity as a function of temperature over different catalysts: (**a**) Pt/S-1, (**b**) Pt/hS-1, (**c**) Pt/hZSM-5 and (**d**) Pt/cZSM-5 catalysts (MCYP: methylcyclopentane, CYH: cyclohexane, MOCYH: methoxycyclohexane, CYA: cyclohexanol, 2-MOCYA: 2-methoxycyclohexanol, and others: Mainly, cyclohexanone for (**a**,**b**) and cyclopentane for (**c**,**d**)).

The product selectivity versus reaction temperature over different Pt catalysts was further analyzed. As shown in Figure 8, similar product distributions were obtained over the Pt/S-1 and Pt/hS-1 catalysts. 2-methoxycyclohexanol (2-MOCYA) and cyclohexanol (CYA) were the main products. Small amounts of methoxycyclohexane (MOCYH) and cyclohexane (CYH) were also found in the products. As the temperature increased from 220 °C to 280 °C, the selectivity to 2-MOCYA gradually decreased from 86% to 59% for the Pt/S-1 catalyst and from 87% to 65% for the Pt/hS-1 catalyst. While the selectivity to CYA significantly increased from 7% up to 23% for Pt/S-1 and 24% for Pt/hS-1 with a little increase to MOCYH and cyclohexanone. This indicated that for the Pt/S-1 and Pt/hS-1 catalysts high temperature would facilitate the cleavage of methoxy group (-OCH_3_) and hydroxy group (-OH) of 2-MOCYA to form CYA and MOCYH as well as dehydrogenation of CYA to cyclohexanone. Moreover, cyclohexene, cyclopentane (CYP), and methylcyclopentane (MCYP) were also detected at high temperature (280 °C), which came from the further reaction of CYH on metal active sites. The selectivity to complete deoxygenated product CYH over Pt/S-1 was a little higher than that of Pt/hS-1. This could be attributed to the higher acid site density of Pt/S-1 catalyst and the high mesoporosity of Pt/hS-1, which facilitated the diffusion of guaiacol and intermediates resulting in lower selectivity to deoxygenated products. These results indicated that S-1 and hS-1 supported Pt catalysts have strong hydrogenation ability and Pt metal sites were responsible for hydrogenation of aromatic rings in guaiacol, agreeing with the literature [64].

Interestingly, the Pt/hZSM-5 and Pt/cZSM-5 catalysts exhibited significantly different product distributions compared to Pt/S-1 and Pt/hS-1 catalysts. Guaiacol was completely transformed into cycloalkanes over Pt/hZSM-5 at the range of experimental temperatures. Compared with Pt/hZSM-5 catalyst, Pt/cZSM-5 catalyst needed a higher temperature to completely transform guaiacol into cycloalkanes (260 °C vs. 220 °C), indicating that the hollow Pt/hZSM-5 catalyst had better deoxygenation ability due to the formation of more Pt active sites adjacent to acid sites in hollow structure. At low temperature of 220 °C, guaiacol was transformed into CYH with a selectivity more than 93% over Pt/hZSM-5, suggesting that this catalyst had strong C-O cleavage ability. As the reaction temperature increased, the selectivity to CYH gradually decreased. And this decrease became significant at 280 °C with the increase of CYP. Under the experimental conditions, CYH could isomerize into MCYP and then demethylate to CYP at high temperature. However, the selectivity to MCYP almost maintained at about 6% as the temperature increased. This indicates that high temperature is prone to the isomerization-demethylation of CYH to CYP over the Pt/hZSM-5. But as for the Pt/cZSM-5 catalyst, the CYH selectivity was only 70% at 220 °C with about 18% of oxygenates, including 2-MOCYA and MOCYH. As the temperature increased up to 240 °C, most of the oxygnetates were converted into CYH and only a trace of 2-MOCYA was found. At high temperature, the Pt/cZSM-5 catalyst showed the similar variation of CYP, MCYP and CYH with Pt/hZSM-5 catalyst. However, a higher selectivity to MCYP and a lower selectivity to CYH were obtained compared with Pt/hZSM-5 catalyst, probably attributed to stronger acidity of Pt/cZSM-5 and the promoted diffusion of products to inhibit the isomerization by the hierarchical porous structure.

**Figure 9:**
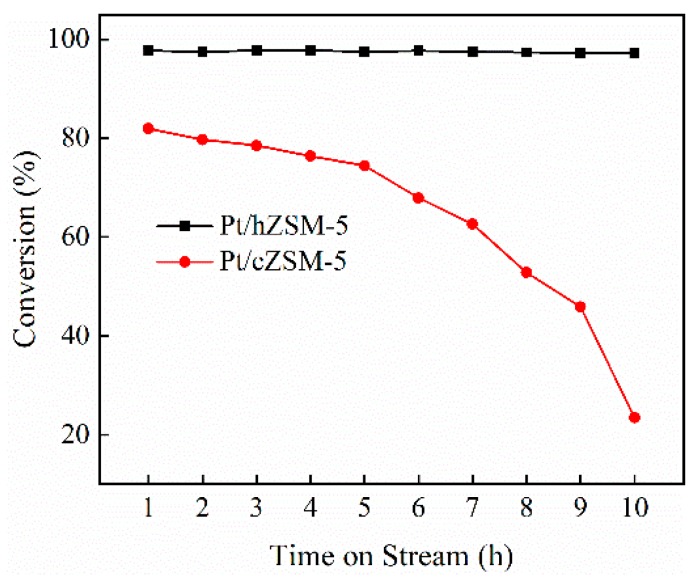
Guaiacol conversion as a function of time on stream over Pt/hZSM-5 and Pt/cZSM-5 catalysts.

**Figure 10:**
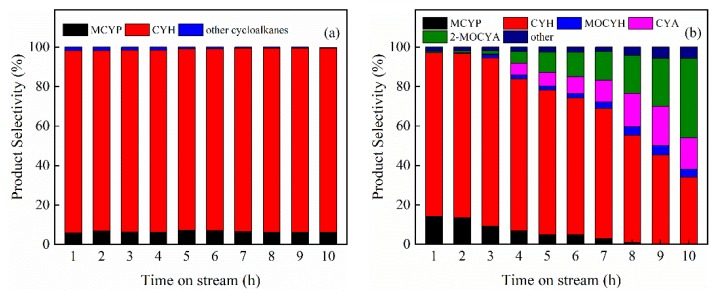
Product selectivity as a function of time on stream over (**a**) Pt/hZSM-5 and (**b**) Pt/cZSM-5 catalysts (MCYP: methylcyclopentane, CYH: cyclohexane, MOCYH: methoxycyclohexane, CYA: cyclohexanol, 2-MOCYA: 2-methoxycyclohexanol).

To further explore the effect of hollow structure on guaiacol HDO, the catalyst stability was also evaluated over Pt/hZSM-5 and Pt/cZSM-5 catalysts at 240 °C under 3 MPa for 10 h. As shown in Figure 9, the guaiacol conversion over the Pt/cZSM-5 catalyst gradually decreased from 82% to 74% in the first five hours, and then decreased drastically, probably due to the coke deposition blocking the pore and covering the active sites of microporous cZSM-5 zeolite. Meanwhile the Pt/hZSM-5 catalyst exhibited excellent long-term catalytic stability. Even at high conversion the Pt/hZSM-5 catalyst showed good resistance to carbon deposition (Figure S3) and the guaiacol conversion had no decrease in the period of experiment. This result demonstrated that the hollow hierarchical structure significantly enhanced the stability of Pt catalyst by promoting the mass transfer of products and reactants. The product distributions in different time on Pt/hZSM-5 and Pt/cZSM-5 catalysts are shown in Figure 10. For the Pt/hZSM-5 catalyst, no significant variation in product distribution was observed and excellent stability was achieved with the selectivity to CYH more than 93%. However, Pt/cZSM-5 showed very poor stability. As the reaction prolonged, the selectivity to total cycloalkanes gradually decreased with the quick increase to oxygenates such as 2-MOCYA, CYA, MOCYH, and cyclohexanone because of the decrease of the acid sites covered by coke deposition to reduce the deoxygenation ability.

## 4. Conclusions

In summary, hollow Silicalite-1 and ZSM-5 zeolites with hierarchical porous structure were synthesized by using the dissolution-recrystallization strategy and exhibited superior surface area and porosity compared with the parent Silicalite-1. The hollow ZSM-5 zeolite supported Pt catalyst exhibited excellent catalytic activity and long-term stability for hydrodeoxygenation of guaiacol. The enhancement of activity and stability could be attributed to the synergetic effect of hollow structure and the interaction of metal-acidic support. The special hollow structures promoted the dispersion of Pt and improved the mass transfer of reactants and products. Therefore, hollow ZSM-5 zeolite supported Pt catalyst showed high activity with 100% selectivity to cycloalkanes, even at a low temperature of 220 °C, suggesting that hollow ZSM-5 zeolite is a promising support for upgrading of bio-oils. This work provides some new insights on designing efficient, highly selective, and stable catalysts for bio-oils upgrading.

## Figures and Tables

**Table 1:** Textual properties of various supports.

Samples	Specific Surface Area (m^2^·g^−1^)			Pore Volume (cm^3^·g^−1^)		
	S_BET_^a^	S_micro._^b^	S_ext._^c^	V_total_^d^	V_micro._^b^	V_meso._^e^
cZSM-5	332.04	218.34	113.70	0.1920	0.1366	0.0554
S-1	408.85	289.98	118.87	0.2411	0.1421	0.0990
hS-1	372.22	142.03	230.19	0.2483	0.0715	0.1768
hZSM-5	386.96	157.88	229.08	0.2585	0.0804	0.1781

^a^ Measured by BET method. ^b^ Determined by t-plot method. ^c^ External surface area was calculated as S_ext._ = S_BET_ − S_micro__._^d^ Total pore volume taken from the volume of N_2_ adsorbed at P/P_o_ = 0.95. ^e^ V_ext._ = V_total_ − V_micro._

**Table 2:** Acidity properties and CO chemisorption of various Pt catalysts.

Samples	Si/Al ^a^	Peak Temperature (°C)			Amount of Acid Sites (μmol·g^−1^)				CO Uptake (μmol·g^−1^)	Dispersion ^b^ (%)
		Peak I	Peak II	Peak III	Weak	Medium	Strong	Total		
Pt/cZSM-5	80	172	232	364	208.03	72.61	144.1	424.74	7.98	15.56
Pt/S-1	—	147	—	329	32.44	—	19.15	51.59	6.79	13.24
Pt/hS-1	—	150	—	—	7.08	—	—	7.08	14.01	27.32
Pt/hZSM-5	175	177	285	380	128.37	43.8	87.75	259.93	17.36	33.86

^a^ The silica alumina ratio determined by ICP-OES. ^b^ The metal dispersion determined by CO chemisorption.

## Supplementary Materials

The following are available online at https://www.mdpi.com/2079-4991/9/3/362/s1, Figure S1: FT-IR spectra of cZSM-5, S-1, hS-1 and hZSM-5 zeolites, Figure S2: ^27^Al NMR spectra of cZSM-5 and hZSM-5 zeolites, Figure S3: TG curves of used Pt/cZSM-5 and Pt/hZSM-5 catalysts after stability tests. Table S1: XPS quantitative data of different Pt catalysts.
