# Supplementary material for: Hollow MFI Zeolite Supported Pt Catalysts for Highly Selective and Stable Hydrodeoxygenation of Guaiacol to Cycloalkanes
Source: Nanomaterials (Basel). 2019 Mar 4;9(3):362. doi: 10.3390/nano9030362 (PMC6473964; doi:10.3390/nano9030362)
Supplement: Supplementary file 1 [file nanomaterials-09-00362-s001.pdf]

Hollow MFI Zeolite Supported Pt Catalysts for Highly Selective and Stable Hydrodeoxygenation of Guaiacol to Cycloalkanes

Xiaopo Niu[1], Fuxiang Feng[1], Gang Yuan[1], Xiangwen Zhang[1,2], Qingfa Wang[1,2,*]

[1] Key Laboratory for Green Chemical Technology of Ministry of Education, School of Chemical Engineering and Technology, Tianjin University, Tianjin 300072, P. R. China

[2] Collaborative Innovation Center of Chemical Science and Engineering (Tianjin), Tianjin University, Tianjin 300072, P. R. China

[*] Corresponding author. Tel. +86-22-2789-2340.

E-mail address: qfwang@tju.edu.cn (Q. Wang).

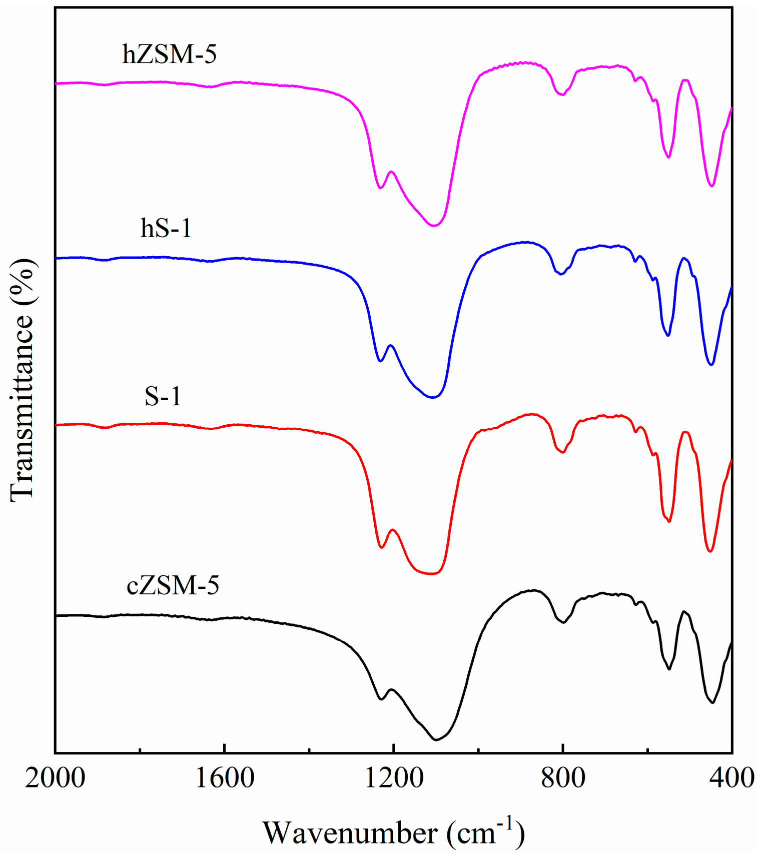

Figure S1 FT-IR spectra of cZSM-5, S-1, hS-1 and hZSM-5 zeolites

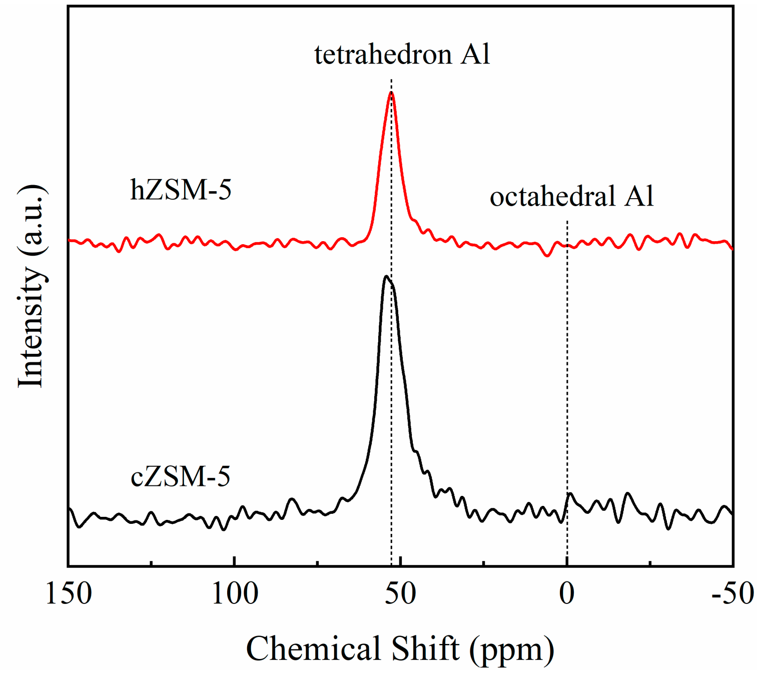

Figure S2 ^{27}Al NMR spectra of cZSM-5 and hZSM-5 zeolites

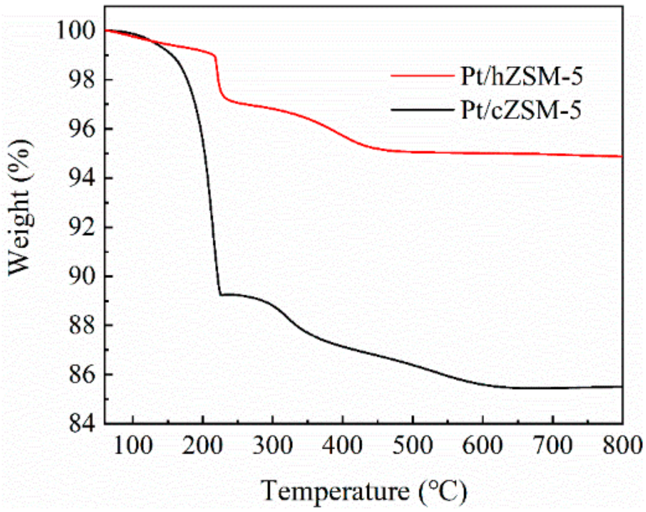

Figure S3 TG curves of used Pt/cZSM-5 and Pt/hZSM-5 catalysts after stability tests

The thermogravimetric analysis (TG) curves of used Pt/cZSM-5 and Pt/hZSM-5 catalysts after stability tests are shown in Figure S3. The amount of deposited carbon is 5.0 wt% and 14.5 wt% for used Pt/hZSM-5 and Pt/cZSM-5 catalysts, respectively. This indicated that the Pt/hZSM-5 catalyst showed good resistance to carbon deposition even at high conversion. In addition, this also indicates that the deactivation of the Pt/cZSM-5 catalyst is mainly due to carbon deposition blocking the pore and covering the active sites of microporous cZSM-5 zeolite.

Table S1 XPS quantitative data of different Pt catalysts

Catalyst	Pt 4f 7/2		Pt 4f 5/2	
	Position (eV)	Peak Area	Position (eV)	Peak Area
Pt/S-1	71.65	2522	74.95	1891
Pt/hS-1	71.76	1922	75.06	1442
Pt/hZSM-5	71.83	990	75.13	743
Pt/cZSM-5	71.80	1144	75.10	858
